# CT angiography of acute aortic syndrome in patients with chronic kidney disease

**DOI:** 10.1007/s10554-025-03336-7

**Published:** 2025-01-24

**Authors:** Angeliki Papachristodoulou, Patrick Ghibes, Natalia Valeria Pentara, Maria Alexandratou, Abraham Levitin, Sameer Gadani, Sasan Partovi, Elizabeth Psoma, Vasileios Rafailidis, Panos Prassopoulos

**Affiliations:** 1 Department of Clinical Radiology, AHEPA University Hospital of Thessaloniki, Aristotle University of Thessaloniki, Thessaloniki, Greece; 2 Department for Diagnostic and Interventional Radiology, University Hospital Tuebingen, Tuebingen, Germany; 3 Interventional Radiology, Cleveland Clinic Main Campus, Cleveland, OH USA

**Keywords:** Acute aortic syndrome, Computed tomography angiography, Atherosclerosis, Chronic kidney disease

## Abstract

The term acute aortic syndrome (AAS) refers to a range of different entities, including dissection, intramural haematoma and penetrating atherosclerotic ulcer. Patients with chronic renal disease and particularly those with dominant polycystic kidney disease are susceptible to this pathology, given the underlying renal arteriopathy and hypertension. Imaging plays a crucial role in diagnosing, grading and guiding management of these patients, with computed tomography angiography (CTA) being on the frontline. Albeit of overlapping of imaging findings between these conditions, specific imaging characteristics help discriminate and guide treatment. Given the nephrotoxic contrast agent involved, tailored CTA protocols or alternative imaging modalities such as MRI or US are necessary in this patient population. This review article discusses the main imaging findings of entities found in the spectrum of AAS, as well as the appropriate use and protocol of imaging modalities, focusing on the appropriate use of nephrotoxic contrast agents, the preservation of renal function and maintenance of optimal diagnostic accuracy.

## Introduction

Acute aortic syndrome (AAS) encompasses a spectrum of life- threatening entities including aortic dissection (AD) as well as incomplete dissection (ID), intramural hematoma (IMH) and penetrating aortic ulcer (PAU). These entities describe some degrees of loss of integrity of the aortic wall, thereby increasing the risk for aortic rupture and / or end-organ malperfusion, leading to ischemia [1]. AAS are subcategorized based on the degree of involvement of the affected aortic wall [2]. Patients with chronic kidney disease (CKD) are prone to developing AAS due to renal arteriopathy and hypertension. Therefore the appropriate clinical presentation in this patient population should prompt immediate imaging evaluation [3]. Certain subcategories of CKD, particularly dominant polycystic kidney disease are at increased risk for developing AAS secondary to the known gene mediated aortic media layer alterations [3].

In general, patients with AAS are subdivided based on the Stanford classification with regard to associated risk, thereby clarifying subsequent management decisions. In the Stanford A category aortic lesions are included which are affecting the ascending aorta and the Stanford B category encompasses those lesions distal to the origin of the left subclavian artery [4]. Many entities of AAS may coexist in the same patient or develop meta-chronously. They are considered acute within the first 14 days of symptoms onset [5]. Although it is difficult to define the precise incidence of AAS due to the high prehospital admission mortality associated with this disease entity, it occurs in general more common in male patients and the incidence is increasing with advanced age [6]. With regard to prehospital admission mortality, it is estimated that approximately 30–50% of patients with Stanford A aortic lesions do not survive the time from disease onset to hospital admission [2]. AAS can present with a wide variety of symptomatology depending on disease stage and extent and the symptomatology is also dependent on the associated complications. Pathology of the ascending aorta (type A) is associated with severe anterior chest pain, while pathology of the descending aorta (type B) may manifest as severe back pain [1]. Most patients with PAU are asymptomatic and therefore this may be an incidental imaging finding [2].

Recent European and U.S. guidelines recommend a three step diagnostic algorithm for the detection of AAS combing the patient’s pretest probability, EKG, chest x-ray, transthoracic echocardiography (TTE), and d-dimers. Based on the results, patients with high clinical suspicion for AAS should undergo immediate computed tomography angiography (CTA) evaluation which is the modality of choice in the acute setting [2, 7]. Studies have proposed the development of multidisciplinary emergency care teams under a “code acute aortic syndrome” in quaternary medical centers. This would particularly benefit patients with CKD not only by facilitating diagnosis but also by subsequent individualized workup and management, either via tailoring the CTA protocol and contrast agent dose or in selected cases by using alternative modalities, such as CEUS or MRA if clinical picture and symptomatology permits [2].

## CTA protocol and common imaging findings

CTA offers the benefits of a short acquisition time, wide field of view and the modality is widely available. In AAS the use of electrocardiogram (EKG) gating is of utmost importance in order to avoid pitfalls in the area of the aortic root where motion artifacts occur [8]. Gating technology eliminates motion artefacts of the heart as well which can appear as a ‘’double aortic wall’’ or result in a false positive diagnosis of a dissection flap within the ascending aorta [2, 9]Modern scanners with state-of-the-art protocols optimize contrast material administration via decreasing the contrast load while maintaining diagnostic images with uniform enhancement and these protocols have the potential to minimize radiation exposure [10].

A dedicated protocol includes pre-contrast imaging (detection of hematomas, calcifications etc.) and arterial phase imaging using bolus tracking with or without automatic triggering [11]. The optimal threshold ranges between 130 and 150 HU at 100–120 KV depending on the body habitus. The gantry speed should be less than 0.5 s to decrease cardiac motion artifacts. CTA contrast material attenuation can be increased by using tube potentials of 100 kVp, compared with 120 kVp, thereby minimizing the need for higher concentrations and volumes of iodinated nephrotoxic intravenous contrast material [11]. The optimal flow rate is 3 to 5 mL /sec and the optimal lumen opacification can be achieved at 80 kVp settings in patients with lower body weight [12]. A biphasic injection technique is composed of administration of approximately 50 to 60 mL of iodinated contrast, followed by a saline chase of equivalent amount and this technique is particularly helpful in patients with CKD who are benefiting from a decreased amount of the nephrotoxic contrast [13]. In order to achieve a higher contrast-to-noise ratio, injection for chest CTAs in the setting of AAS should be pursued from the right upper extremity as opposed to the left side (if appropriate intravenous access is available on that side) to decrease streak artifacts in the neck and thoracic inlet area which can impact the evaluation of the thoracic aorta [5]. It is important to note that the biphasic injection technique may also be helpful in preventing beam hardening artefacts from contrast opacification of the SVC.

Current contrast dose recommendations are weight based and at least 300 mg iodine contrast per kilogram is recommended to obtain diagnostic images [14]. In specific cases of AAS, delayed venous phase imaging should be acquired in addition to arterial phase imaging in order to differentiate slow flow processes from false lumen thrombosis [15]. When prompt opacification of the aorta is not achieved and/or is inadequate many underlying causes should be taken into consideration, such as intravenous contrast extravasation, low cardiac output, volume overload, morbid obesity as well as wrong ROI positioning during bolus tracking to mention a few. Finally, the field of view should encompass the entire thoracoabdominal aortic course and major arterial branches to the level of the femoral arterial vasculature [16]. In the setting of chest pain, the scan can be aborted after obtaining images of the chest if the thoracic aorta is unremarkable in order to reduce the total radiation dose.

Regarding CT reconstructions, sagittal oblique 45° reconstructions can be helpful for assessment of the aortic arch while coronal reconstructions are useful for assessment of the aortic root. Therefore, coronal and sagittal reconstructions should be routinely pursued for all cases with concern of acute aortic syndrome [10].

### Contrast injection in CKD

The risk of contrast associated acute kidney injury in CKD patients is directly associated with the stage of the disease at the time of contrast administration with the following risks being affected by contrast induced nephropathy: 5% at eGFR greater than or equal to 60, 10% at eGFR of 45–59, 15% at eGFR of 30–44, and 30% at eGFR less than 30 mL/min/1.73 m2. Concomitant nephrotoxic agents, low blood pressure and/ or blood volume, albuminuria and kidney malperfusion (possibly in the setting of congestive heart failure or acute dissection) usually increase the possibility of acute kidney injury in cases of contrast administration.

In patients of AKI or eGFR less than 30 mL/min/1.73 m2 who are not undergoing hemodialysis, prophylaxis is indicated when administering nephrotoxic contrast agents. This includes intravenous isotonic normal saline administration and/ or bicarbonate saline 1 h before the exam continuing 3 to 12 h after the exam with weight-based dosing. In emergency cases though, enough time for prophylaxis might not be available [17]. In CKD stages 4 and 5 contrast should be administered only if risks and benefits have been carefully evaluated and benefits clearly outweigh the risks, for example in case of symptoms concerning for AAS or in case of life threatening bleeding when CTA is warranted to localize the bleeding source and to assess for contrast extravasation in arterial phase imaging [18].

Carefully modifying the CTA protocol in the chronic kidney failure population is essential to minimize the amount of administered contrast. Alternative modalities should be considered and include contrast-enhanced ultrasound (CEUS) and MRA with or without gadolinium-based contrast agents. The decision to use CEUS or MRA relies on the type and extent of disease as well as local resources and expertise. For instance, a localized single abdominal atherosclerotic ulcer initially diagnosed with CTA, could potentially be followed-up partly with CEUS, alternating with CTA or MRA in order to minimize exposure to nephrotoxic contrast agents. Alternative imaging options including protocol considerations for evaluation of acute aortic syndrome are summarized in Table 1.

**Fig. 1:**
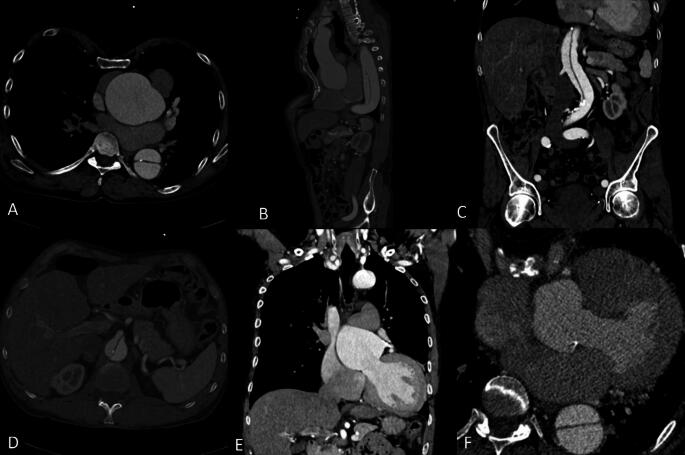
Type B aortic dissection. Axial (**A**) and sagittal (**B**) images depict the dissection membrane in the thoracic aorta beginning at the level of the mid descending aorta. The dissection membrane extends into the proximal right common iliac artery (**C**). Axial image at the level of the mesenteric vessels reveals an origin of the superior mesenteric artery from the true lumen (**D**). Dual source technology of the photon-counting scanner allows almost motion artifact free images of the heart and ascending aorta. In addition to the aortic dissection type B, an ascending aortic aneurysm (**E**) and calcifications of the aortic valve (**F**) are visualized

The recent introduction of photon counting CT in the clinical arena has the potential to further decrease contrast amount while maintaining diagnostic imaging and therefore this technology offers distinct advantages for patients with CKD [19]. Photon-counting CT has shown to improve the iodine signal at the same tube voltage compared to energy-integrating Detectors (EID) of previous CT technology generations [20]. The prospective study of Higashigaito et al. reveals a 25% reduction of iodine contrast when conducting photon counting CTA of the aorta while preserving image quality compared to EID CT scanners [21]. Virtual monoenergetic image reconstructions with 40 keV near the k-edge of iodine allows further reduction of iodine contrast [22]. Another study by Rau et al. on photon counting CTA of the aorta accomplished a 50% reduction of iodine contrast without limitations in diagnostic confidence [23]. Patients with impaired kidney function and the need for repeated exams after acute aortic syndrome may benefit from the reduced contrast media dose required for photon counting CT exams (Fig. 1).

### The role of MRI

**Fig. 2:**
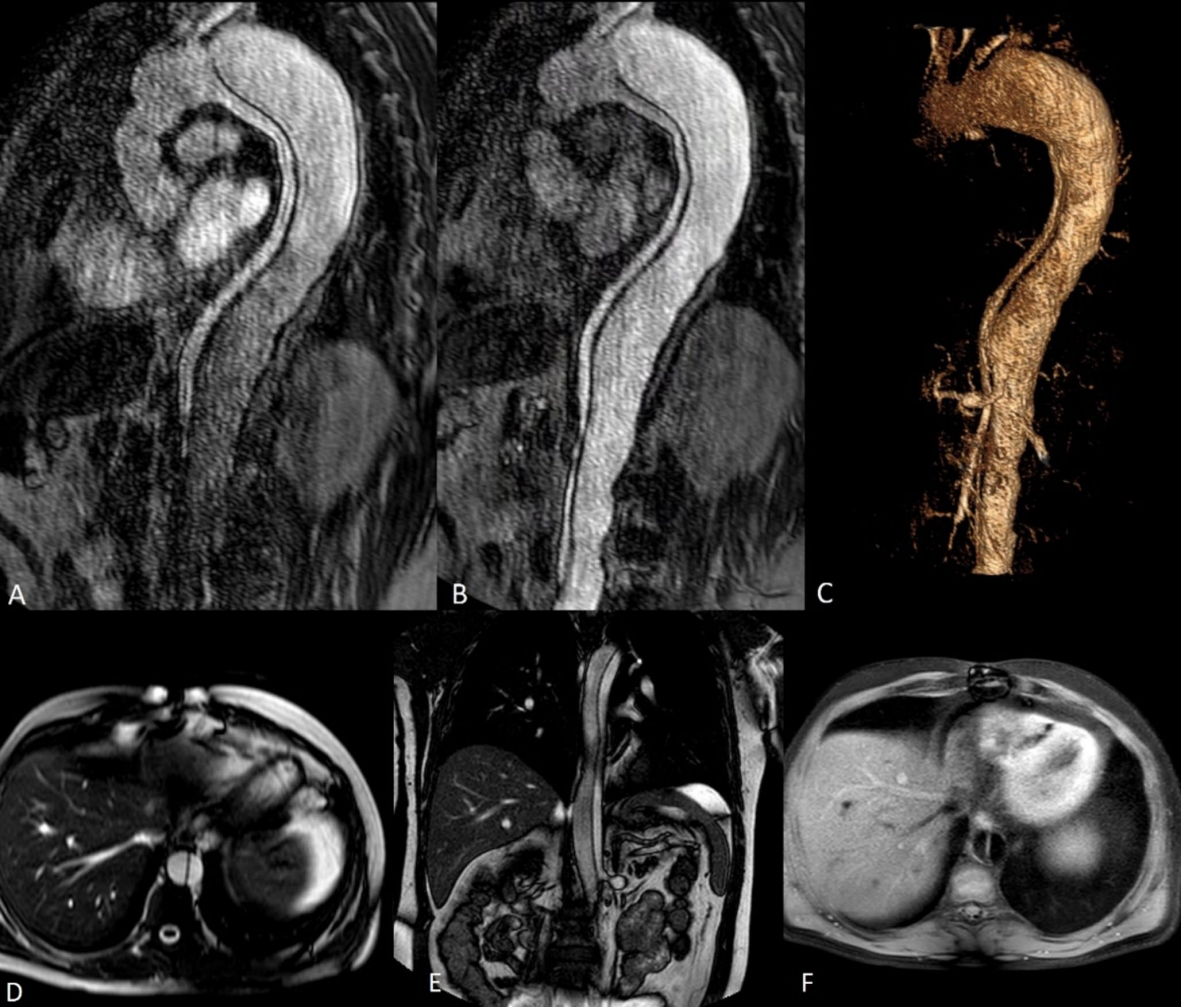
Follow-up contrast-enhanced MRA of patient with known type B aortic dissection MRA. Sequential dynamic MRA images in the arterial phase enable dynamic evaluation of the true and false lumen hemodynamics including visualization of slow blood flow (**A**, **B**). Volume rendered image showing the full extent of the dissection and the origins of the main aortic branches (**C**). The intimal flap is well visualized in T2 weighted (**D**, **E**) and T1 weighted (**F**) images

CTA is widely available, particularly in the emergency department setting. However, patients with CKD may benefit from the use of MRI in certain scenarios, thereby avoid administration of nephrotoxic iodinated contrast. MRA may also be useful in the follow-up setting of AAS to detect potential complications in patients with underlying CKD [24]. Steady-state free precession (SSFP) sequences can contribute to the localization of the intimal media flaps in AAS, entry and re- entry points, exit tears, and / or floating acute thrombi. SSFP has been shown to be more sensitive than 3D CE-MRA with regard to the detection of these findings and the exam can be completed in a relatively short time frame which is relevant in the critically ill AAS patient population [24, 25]. On the other hand, 3D CE-MRA is particularly helpful in the assessment of branch vessel involvement, certain intraluminal pathology, PAU, and other characteristics of dissection flaps in AAS [26] (Fig. 2). Based on the authors’ experience on MR for AAS T1 sequences can be helpful for IMH detection while T2 sequences facilitate evaluation for pericardial effusion or cardiac wall edema in the setting of possible underlying cardiac ischemia.

## Aortic dissection

Aortic dissection (AD) comprises roughly 70–80% of AAS cases and CTA exhibits an excellent sensitivity and specificity of 98 to 100% respectively in diagnosing this disease entity [6]. The pathogenesis includes a tear within the aortic wall separating the intima and medial wall layers and thereby creating a false lumen in the media. In the case of AD a dissection flap, two aortic lumens (true and false lumen) as well as an entry tear are present [7]. Predisposing factors leading to medial aortic wall degeneration include connective tissue disorders like Marfan syndrome, hypertension and pre-existing aortic aneurysms [27]. The CKD patient population is at increased risk for AD due to the higher incidence of arterial hypertension [16]. Stanford type A aortic dissections require urgent surgical repair and outcomes are generally dependent on the extent of complications at the time of disease detection [16]. A more rare subtype of AD is incomplete dissection otherwise called intimal tear without hematoma [28], which is caused histopathologically by a laceration in the intima and media and/or a partial thickness tear without creation of a complete false lumen [7]. Regarding classification it is worth mentioning the non-A non-B dissection with an entry point located neither in the ascending nor the descending aorta, but in the aortic arch [29]. Complications of AD include multiorgan failure due to ischemia, stroke, myocardial infraction, pericardial tamponade due to hemorrhagic pericardial effusion, leg ischemia as well as acute aortic regurgitation due to extension of the AD to the level of the aortic valve [30].

**Fig. 3:**
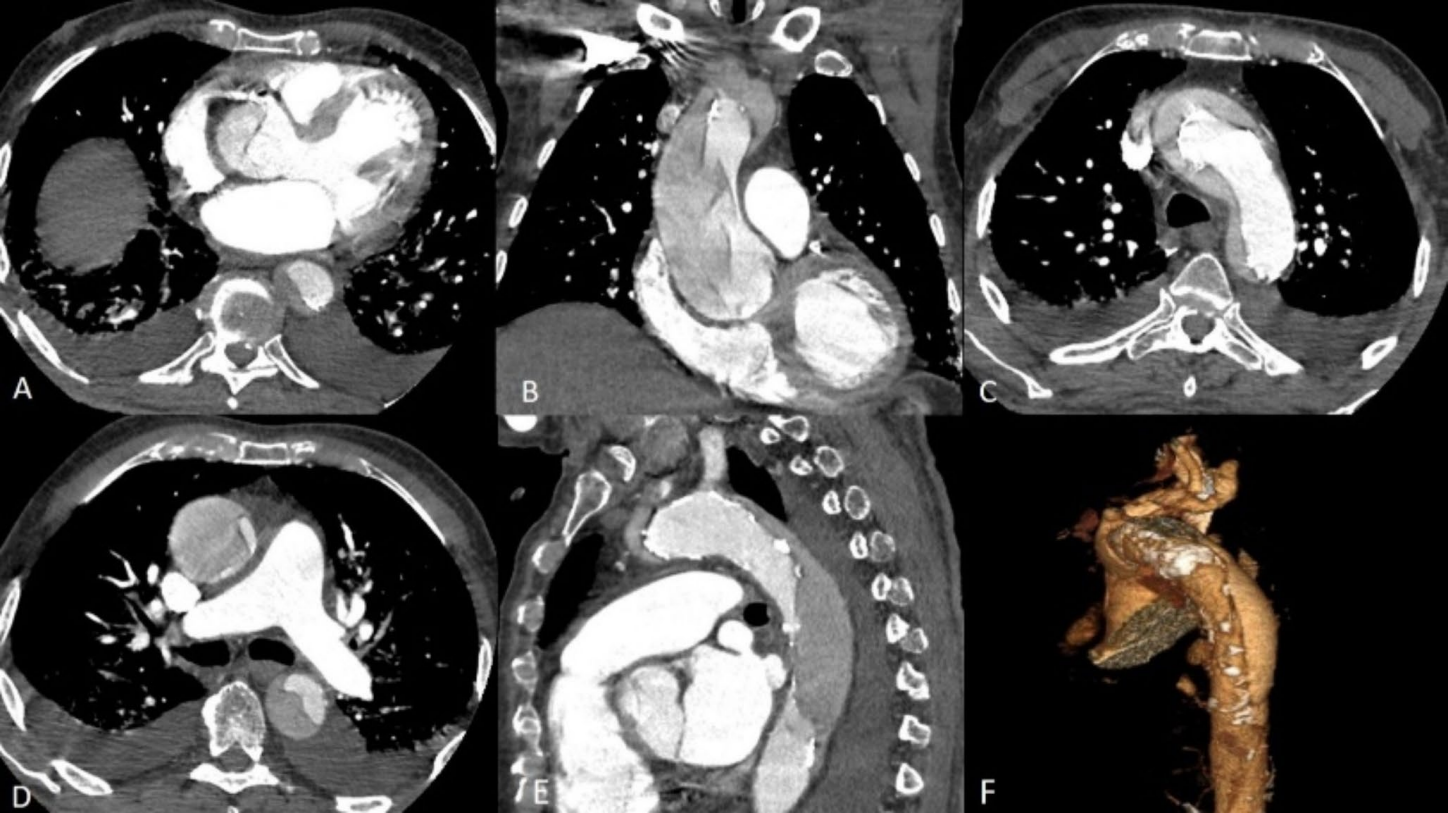
Type A aortic dissection. Axial CTA images at the level of the right coronary artery (**A**), at the level of the aortic arch (**B**) and at the level of the pulmonary artery (**C**) demonstrating the type A aortic dissection. Coronal (**D**) and sagittal (**E**) images reveal the extent of the dissection as it tracks longitudinally along the media layer of the aorta, thereby forming a second blood-filled channel (false lumen) within the vessel wall. Post processing with volume rendered image of the aortic dissection (**F**)

In suspected AD a CTA should include a pre-contrast scan from the level of the neck to the bilateral proximal femurs [31]. Diagnostic suspicion for a Stanford type A AD is occasionally reached based on TEE findings, however the subsequent disease extent and further characterization needs to be pursued with CTA (Fig. 3) [2]. On CTA a low attenuation possibly partially calcified straight or curved dissection flap can be seen in the lumen of a dilated aorta. Reporting of AD should include the exact location, extent, size and thrombosis of the false lumen, as well as the presence of multiple entry points and calcifications. Further, it is important to describe which vessels arise from the true versus false lumen to risk stratify for possibility of organ malperfusion. The size of the true and false lumens varies depending on the cardiac cycle phase image acquisition and typically the true lumen is smaller and starts at the aortic root level [10]. The larger the false lumen the more urgently endovascular or surgical intervention should be pursued in order to preserve blood flow through the true lumen and avoid multiorgan ischemia [32]. Sometimes the two lumens remain patent and communicate through several fenestrations. Ruptures into the pericardium, pleura or peritoneum may happen and can be life threatening [33]. Furthermore, the degree and extent of thrombosis of the involved vessels is associated with severity outcomes like end organ ischemia. Vessel obstruction is subdivided into static (owed mostly to thrombosis) and dynamic (transient stenotic disease / obstruction due to flap movement) [34]. Significant aortic dilatation compared to prior available cross-sectional imaging should be carefully evaluated for due to the high risk of rupture [10]. It is important to consider the acquisition of delayed phase images in addition to the arterial phase to better appreciate affected end organ perfusional defects and the extent of false lumen thrombosis [35]. Imaging findings with significant implications for patient management include Stanford type A vs. B AD, entry point location, presence and extent of pericardial effusion, vessel rupture, and signs of end organ malperfusion [2].

### Penetrating atherosclerotic ulcer

Penetrating aortic ulcer (PAU) of the thoracic aorta was first described by Shennan in 1934 and his hypotheses were essentially confirmed by Stanson et al. in 1986. PAU is the result of an atherosclerotic plaque progression which ulcerates and perforates the internal elastic lamina into the aortic media resulting in hematoma formation within the aortic wall [36]. PAU is a poorly understood entity that accounts for 2–7% of AAS cases [37]. It is a pathologic process confined to the intima, while AD and IMH are disease entities affecting the media of the aortic wall [9]. PAU is a focal lesion that may progress to IMH without an intimal flap due to the media being exposed to pulsatile blood flow and due to erosion of leaking vasa vasorum by the ulcer [38, 39]. This causes essentially a localized AD in which the ‘’crater’’ constitutes the entrance tear [40]. Penetration into the adventitia may lead to a saccular pseudoaneurysm or even rupture which has been reported in up to 38% of PAU cases. Rupture into the mediastinum or pleural cavity has been reported in rare circumstances and thrombus or atherosclerotic ulcer fragments embolizing distally may occur rarely with PAU [2, 9, 37, 39].

Concerning the natural history of this lesion, it is frequently associated with localized thickening of the aortic wall, diffuse surrounding atherosclerotic disease and extensive aortic calcifications. Along the final pathway of the other AAS sub-entities medial degeneration takes place as the disease progresses. Although PAU can be observed in an aorta of normal diameter, it is commonly found in aortas aorta with enlarged diameter, particularly in those patients who have already developed thoracoabdominal aortic aneurysms [2, 38–40]. It can potentially present anywhere along the length of the aorta but the most typical segments are the mid and distal descending thoracic aorta (type B PAU) followed by the ascending (type A PAU) and abdominal aorta. PAU can be multifocal since atherosclerotic disease is the major risk factor of PAU and atherosclerosis typically manifests as multifocal disease throughout the thoracoabdominal aorta [9].

Patients with advanced age (> 60 years) and advanced aortic atherosclerotic disease are at increased risk for developing ulceration of an atherosclerotic plaque. Important comorbidities in this patient population include poorly controlled arterial hypertension, diabetes, COPD, cardiac and renal failure. In addition, accelerating factors for the disease involve smoking and hyperlipidemia. When the integrity of the aortic wall is affected by a pathologic process in a young normotensive patient without atherosclerosis, underlying connective tissue disorders, a vascular inflammatory process or trauma should be taken into the consideration as underlying pathophysiologic processes for the development of PAU [1, 39, 40].

**Fig. 4:**
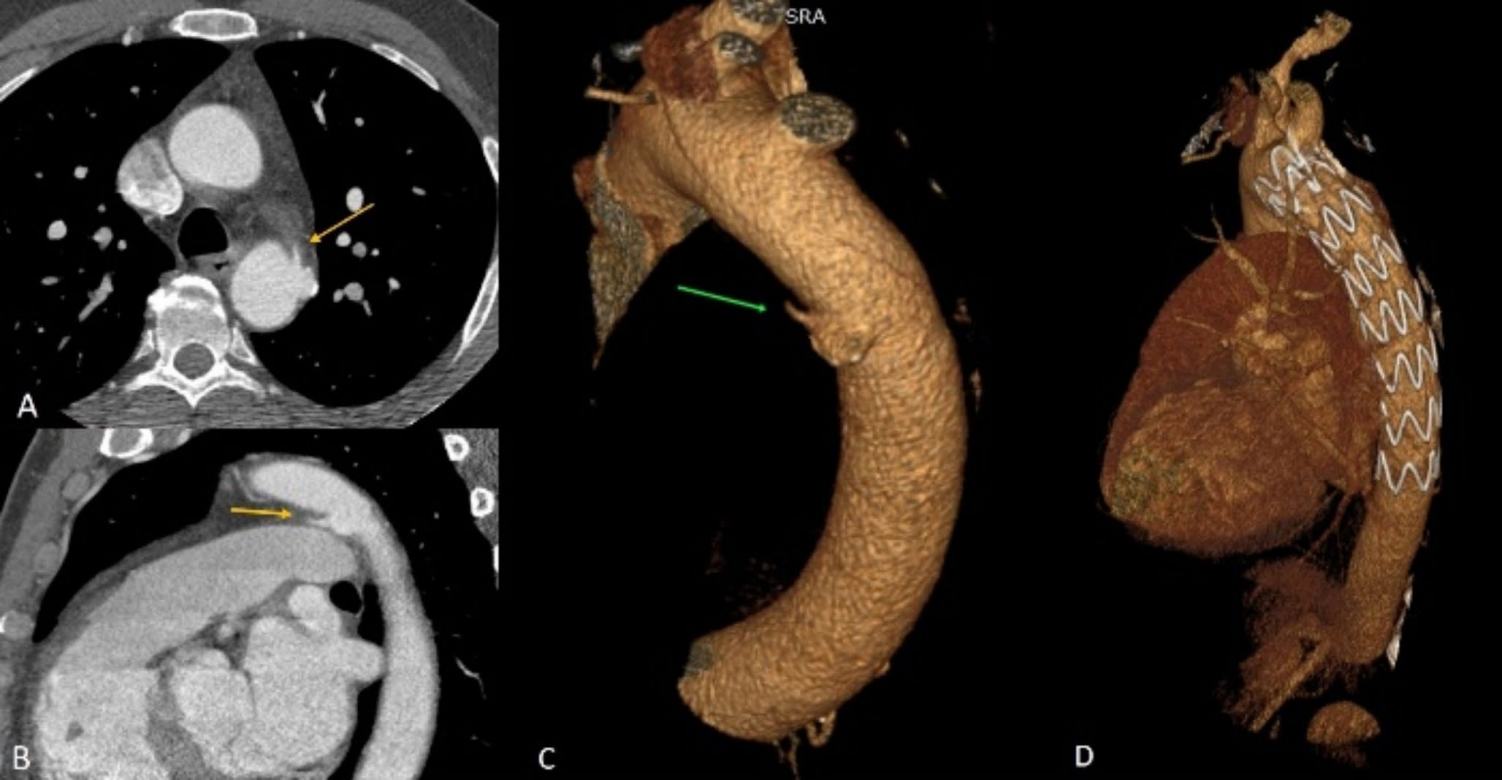
Penetrating aortic ulcer. Axial (**A**) and sagittal (**B**) computed tomography angiography (CTA) images of a thoracic penetrating aortic ulceration at the distal aortic arch / most proximal descending aorta (yellow arrow). Volume rendered images of the thoracic aorta with the ulceration pre-intervention (**C**) and post repair with TEVAR (**D**). Post intervention images showing no evidence of residual penetrating aortic ulceration

Regarding diagnostic imaging, transoesophageal echocardiogram (TEE), transthoracic echocardiogram (TTE), computed tomography angiography (CTA) and magnetic resonance imaging angiography (MRA) can all potentially diagnose PAU along with its complications [9]. Computed tomography angiography (CTA) is the imaging modality of choice in the emergent setting due to its wide availability and rapid acquisition techniques (Fig. 4) [1, 2, 37].

**Fig. 5:**
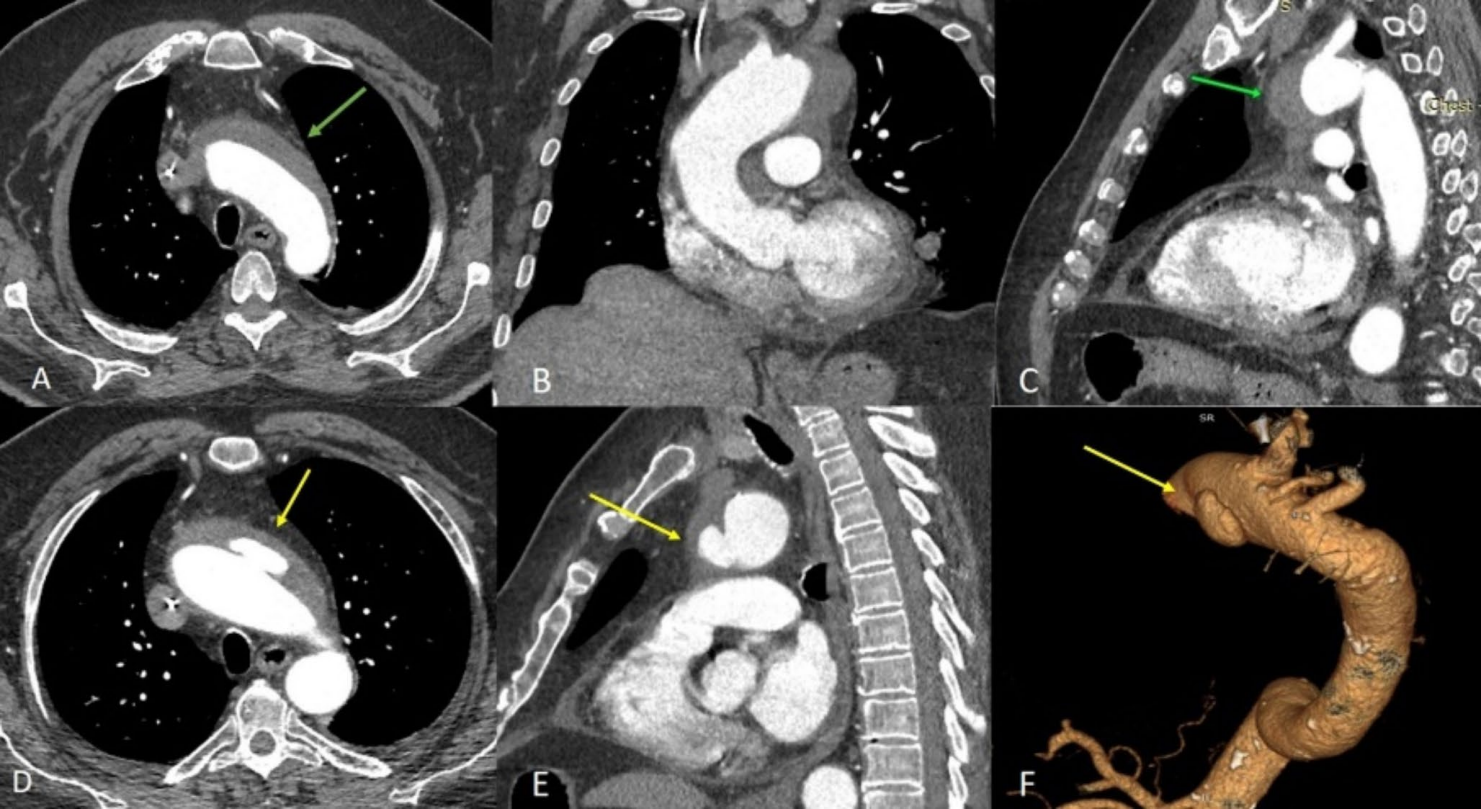
Intramural hematoma with an ULP (ulcer-like projection). Axial (**A**) coronal (**B**) and sagittal (**C**) computed tomography angiography (CTA) images of an intramural hematoma of the thoracic aorta (green arrows). Axial (**D**) and sagittal (**E**) images of the thoracic aorta at the site of the coexisting ULP. Post processing with volume rendered image of the ULP (**D**)

PAU can be acute or chronic in nature. Acuity is implied when an IMH or signs of contrast extravasation are present [41]. On unenhanced CT, PAU may appear similar to an IMH. Contrast enhanced CT demonstrates a craterlike or ulcer-like outpouching of the lumen within the aortic wall with possible inward displacement of the calcified tunica intima by the IMH or localized thickening of the vessel wall together with a focal outpouching of the outer aortic silhouette (Fig. 5) [38, 41]. The projection of the ulcer past the intima into the medial aortic layer together with the focal silhouette change may distinguish PAU from the common chronic atheromatous ulcer [42].

According to the American Society of Echocardiography and the European Association of Cardiovascular Imaging guidelines, once PAU is detected, it is important to evaluate (1) the maximum depth of perforation of the ulcer from the aortic lumen along with location; (2) its maximum width at the entry site; (3) the axial length / extension of the related hematoma, if present; (4) contrast extension beyond the aortic wall and (5) the length of a false lumen, if present [9]. Calculating the depth and diameter of the ulcer can be used for risk stratification purposes, namely PAUs with a diameter over 13 to 20 mm and a depth above 10 mm have been correlated to unfavorable outcomes and therefore patient management needs to be adjusted accordingly in these higher risk patients [1, 38].

It is important to acknowledge that PAU, ulcer like projections (ULPs) and intramural blood pools (IBPs) are distinct terms. ULPs are believed to be the result of new intimal disruption in the area of an IMH. Their appearance on imaging is a small localized blood filled pouch protruding from the aortic lumen into the aortic wall or from the true aortic lumen into the thrombosed false one with a wide communicating neck which is measuring above 3 mm [43]. The most common location of a ULP is in the descending thoracic aorta (68%), followed by the distal aortic arch (13%) and ascending aorta (18%) [10]. Unlike PAUs ULPs are not associated with atherosclerotic plaques but are characterized by a smooth luminal surface. IBPs, also known as aortic branch artery pseudoaneurysms are small blood pools within an aortic intramural hematoma. On contrast enhanced CTA they appear as small rounded areas of contrast enhancement within an IMH and a pinhole communication (< 2 mm) with the aortic lumen through the ostia of the intercostal, lumbar or less frequently the bronchial arteries may be visualized on cross sectional imaging [2, 44].

**Fig. 6:**
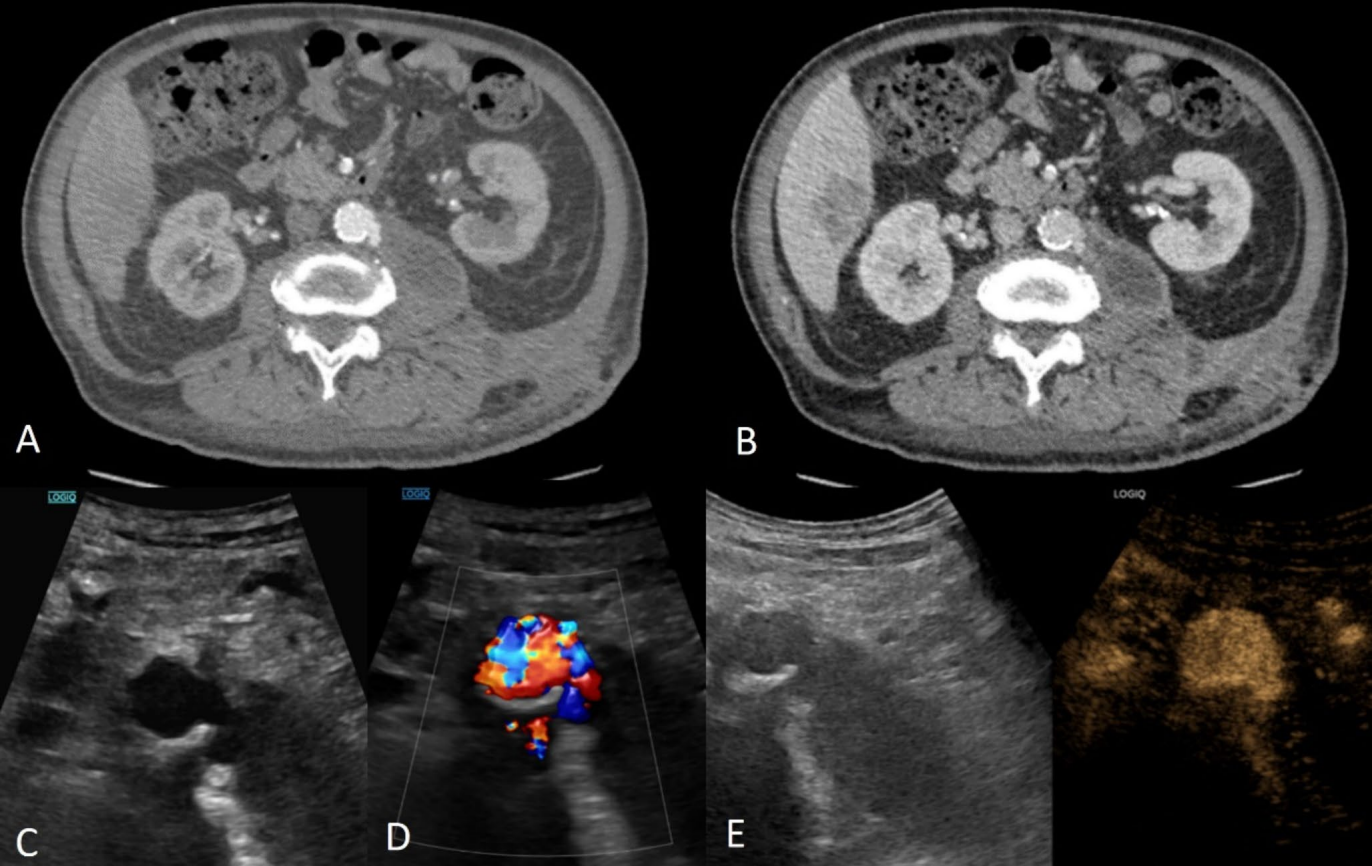
68 year-old male with penetrating atherosclerotic ulcer in the abdominal aorta. Arterial (**A**) and venous phase (**B**) CT images showing a focal outpouching of the contrast agent at the level of the infrarenal abdominal aorta. Note the abutting collection within the psoas muscle, probably related to subacute hemorrhagic leaking. There is no pooling of the contrast agent on the venous phase image and therefore constellation of findings are consistent with a penetrating atherosclerotic ulcer. This aortic pathology was followed-up with US including CEUS. B-mode (demonstrating a predominantly hypoechoic lesion causing discontinuity of the aortic wall (**A**). Color Doppler technique revealed flow reversal inside the penetrating aortic ulcer. CEUS demonstrates enhancement of the lesion with contrast microbubbles but confidently excludes contrast extravasation to suggest active hemorrhage at the time of the exam since there is no contrast opacification appreciated outside of the penetrating ulcer contours or within the abutting psoas collection

Yucel et al. demonstrated that MRI could be more useful than CT at differentiating acute intramural hematoma from atheroma and chronic intraluminal thrombus secondary to the superior tissue characterization properties of MRI [45]. On echocardiography, both transthoracic and transoesophageal findings include a crater like projection of the aortic wall, mostly associated with an abutting atherosclerotic plaque. Similar findings can be seen in the abdominal aorta using conventional B-mode or contrast-enhanced ultrasound (Fig. 6).

The 2014 ESC guidelines on the diagnosis and treatment of aortic diseases stated recommendations on PAU management including the prevention of rupture and the prevention of disease progression to acute AD. Severe pain and signs of contained rupture on imaging indicate the need for urgent intervention. Pain relief and blood pressure control is recommended for all patients with PAU. In type A PAU the possibility of surgery should be explored. In the case of uncomplicated type B PAU careful surveillance including follow-up imaging is recommended while in complicated type B PAU TEVAR or open surgical approaches should be strongly considered [46].

### Intramural aortic hematoma

Intramural aortic hematoma (IMH) is a condition characterized by the presence of hemorrhage within the wall of the aorta without an intimal tear which distinguishes this entity from a classic aortic dissection. IMH are reflective of an acute process and may occur spontaneously or post traumatic [47].

In chronic renal failure patients the risk of acute aortic syndrome and specifically IMH is increased. The main reason for this is the often accompanying chronic hypertension in these patients resulting in continuously elevated pressure exposure of the aortic wall, thereby leading to increased susceptibility to intramural bleeding [48]. Moreover, the uremic state and dysregulation of electrolytes promote accelerated calcific atherosclerotic processes resulting in impaired vascular compliance which in turn predisposes the affected thoracoabdominal aorta to tears and injury of the vasa vasorum [49]. Particularly end stage renal disease patients on hemodialysis are affected by hemodialysis related hemodynamic fluctuations and shifts in intravascular volume which can contribute to an increased risk of IMH formation [50].

The primary event of intramural bleeding is the rupture of the vasa vasorum which are small blood vessels that supply the outer portion of the walls of large arteries including the aorta. The leakage of vasa vasorum leads to accumulation of blood products within the media layer of the aortic wall, thereby creating an intramural hematoma [51]. The hematoma can expand both circumferentially and / or longitudinally which is causing progressive separation of the aortic wall layers. In certain cases IMH may stabilize in size without further complications and gradually resolve over time [52]. However, if the IMH results in an intimal tear, the disease entity may progress to an aortic dissection or even weaken the aortic wall enough to cause a life-threatening aortic rupture [53].

Patients with IMH usually present with sudden onset of severe chest or back pain and the symptoms may mimic myocardial infarction or aortic dissection. Given the non-specificity of symptomatology early cross-sectional imaging is critical performing CT angiography or MR angiography for accurate diagnosis and effective individual patient management [54].

**Fig. 7:**
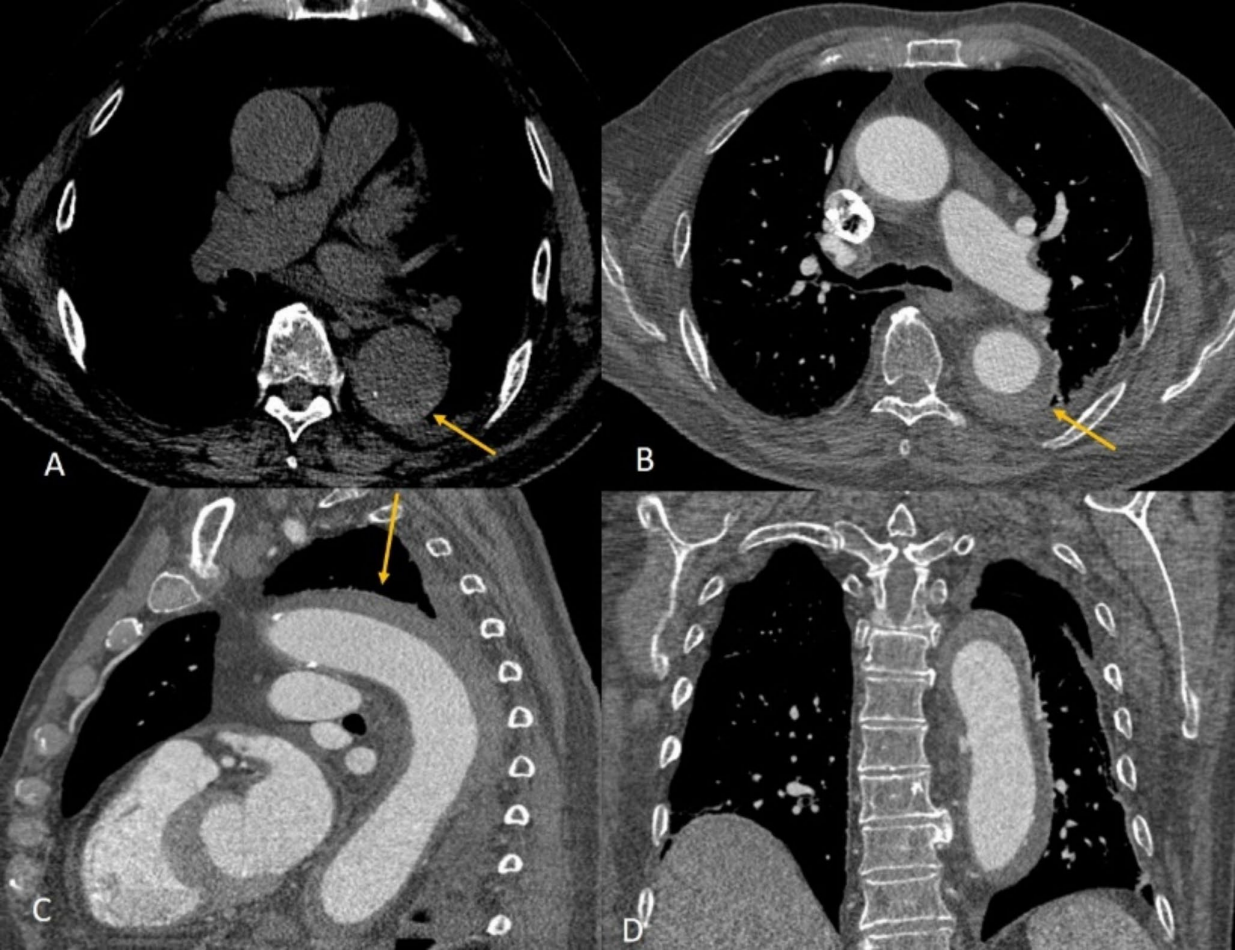
Intramural hematoma. Non-enhanced axial CT image depicts a near-circumferential high attenuation soft tissue area (yellow arrow) within the wall of a dilated descending aorta. The blood in the lumen of the aorta is lower in attenuation compared to the aortic wall on this non-contrast image. There are also intimal calcifications as this type of AAS is considered an atypical form of aortic dissection due to hemorrhage into the aortic wall from leaking vasa vasorum without an intimal tear (**A**). Axial contrast enhanced CT angiography image shows opacification of the aortic lumen with persistence of the abnormal circumferential soft tissue within the aortic wall (yellow arrow) (**B**). Sagittal (**C**) and coronal (**D**) CT angiography images demonstrate the extent of the intramural hematoma

CTA imaging is the modality of choice for the diagnosis of aortic intramural hematoma due to its high spatial resolution and its efficiency in the emergency setting [55]. In order to detect IMH it is very important to obtain native images prior to contrast administration. On non enhanced CT (NECT) IMH typically appears as a crescent shaped high-attenuation (densities ranging from 40 to 70 Hounsfield units) area within the aortic wall. The aortic wall may appear irregular and thickened, usually greater than 7 mm aortic wall diameter (Fig. 7) [56]. This high attenuation is related to acute blood products collecting inside the media layer of the aorta [57]. IMH typically does not exhibit contrast uptake since it is determined as a collection of hemorrhagic products confined to the media layer which does not communicate with the true aortic lumen. The hematoma can extend longitudinally along the aorta and may involve the ascending thoracic aorta, aortic arch, descending thoracic aorta, and abdominal aorta. The length of the IMH is variable. The true aortic lumen will enhance with contrast, thereby helping to delineate the extent of the IMH within the aortic wall [8].

Unlike in the case of aortic dissection, in IMH the intima layer of the thoracoabdominal aorta remains intact. This lack of an intimal flap differentiates IMH from a true aortic dissection on imaging [58]. Calcifications can help differentiate chronic changes in the aortic wall from acute intramural hematoma. There may also be evidence of small amount of hemorrhagic products in the periaortic region, typically visualized as high density fat stranding surrounding the vessel. In more severe cases of IMH associated hemorrhagic pericardial effusion or in cases of involvement of the ascending thoracic aorta left sided hemorrhagic pleural effusion can be detected [59]. CTA is helpful in surveillance imaging of IMH and to rule out progression to aortic dissection [60].

MRA has been demonstrated to be valuable for diagnosing and monitoring intramural aortic hematoma due to its ability to characterize the composition of the hematoma over time [61]. In the acute phase (first few days) the intramural hematoma typically appears as a high signal intensity area on T1-weighted images due to the presence of methemoglobin within the hematoma. As the hematoma evolves the signal intensity on T1-weighted images may alter as it reflects the breakdown of blood products [62]. On T2-weighted images, the hematoma typically appears as a high signal intensity area with hyperintensity in the periaortic region due to edematous changes. As the hemoglobin is degrading, the signal characteristics can vary. In addition, black-blood MRI techniques such as double inversion recovery suppressing the flowing blood signal can be useful for the delineation of the extent of the hematoma [63]. Furthermore, MRA is helpful in confirming the absence of an intimal tear which differentiates IMH from a true aortic dissection [64].

## Conclusion

The chronic kidney disease patient population is at increased risk for the development of acute aortic syndrome due to the underlying pathophysiologic mechanisms including hypertension and renal arteriopathy leading to increased vascular wall predisposition to tears in the setting of accelerated calcific atherosclerotic disease. Imaging is essential to establish the diagnosis and classification of the acute aortic syndrome. In the chronic kidney disease population imaging workup for AAS needs to be specifically tailored. Modified CTA protocols with decreased amount of nephrotoxic contrast agent administration are required. Alternative modalities, specifically MR angiography and CEUS should be considered in CKD patients, particularly for follow-up after the diagnosis has been established. Future guidelines for the workflow and management of acute aortic syndrome in chronic kidney disease are warranted to optimize outcomes in this complex patient population.

**Table 1:** Alternative imaging modalities and protocols for assessment of acute aortic syndrome in the chronic kidney disease patient population

Considerations in patients with CKD requiring imaging for AAS Alternative options:	
Modality	Comments
Contrast-enhanced Ultrasound	Can be useful for isolated abdominal aortic pathology, enabling accurate diagnosis of intraluminal thrombus, dissection, penetrating atherosclerotic ulcer or aortic rupture.
	Add valuable information of hemodynamics given the high temporal resolution.
	Can be used for follow-up of known aortic pathology, after initial CTA evaluation in order to reduce amount of nephrotoxic contrast agent over time.
CTA	Lower iodine concentration agent and lower contrast volume protocols.
	Lower kV (80 or 100 kV).
	Biphasic injection technique with normal saline chaser.
	Prophylaxis with intravenous sterile normal saline prior and after the CTA examination is effective for nephroprotective purposes as shown in the PRESERVE trial [65].
MRA	Non-contrast MRA sequences may be applied.
	CE-MRA can be used if appropriate, particularly newer Gadolinium based contrast agents are deemed safe for patients with underlying kidney disease.

## Data Availability

No datasets were generated or analysed during the current study.
